# Editorial: Microbial solutions for restoring depleted soils

**DOI:** 10.3389/fmicb.2026.1916545

**Published:** 2026-07-15

**Authors:** Samina Mehnaz, Izzah Shahid, Abhishek Walia, Muhammad Zahid Mumtaz

**Affiliations:** 1 Kauser Abdulla Malik School of Life Sciences, Forman Christian College (A Chartered University), Lahore, Pakistan; 2 Institute of Multidisciplinary Research in Applied Biology, Public University of Navarra, Campus Arrosadia, Pamplona, Spain; 3 Department of Microbiology, College of Basic Sciences, Chaudhary Sarwan Kumar Himachal Pradesh Agricultural University, Palampur, India; 4 Institute of Molecular Biology and Biotechnology, The University of Lahore, Lahore, Pakistan

**Keywords:** biofertilizer, bioremedaition, compost, microbial solutions, SynComs

Prolonged intensive cultivation, coupled with the widespread application of synthetic chemicals and industrial extraction, has expedited the global degradation of soil resources, posing a significant threat to food security worldwide. Contemporary agriculture is experiencing a critical ecological transition due to the extensive depletion of essential soil nutrients and the decline of soil biodiversity. The application of current synthetic supplements, including nitrogen, phosphorus, and potassium fertilizers, and chemical pesticides and fungicides, offers superficial surges in crop yield and fails to address the underlying physiological and structural decline of soil matrices. Additionally, the continuous application of these chemicals exacerbates long-term degradation through soil acidification, groundwater salinization, and destruction of indigenous ecological networks. Consequently, the current research has shifted its focus to finding biological and eco-friendly solutions to restore depleted soils.

The current Research Topic categorically assembles studies investigating the capacities of beneficial microbes and strategic biological amendments to revitalize degraded soil. These studies reiterate the significance of soil as a living ecosystem and a hub for diverse microbial communities that shape root architecture and plant productivity. This study also focused on the design and composition of biological interventions to demonstrate their efficacy across diverse soil textures and chemical and climatic profiles.

A 4-year longitudinal study by Zhou et al. evaluated the residual impact of a single biochar application on aeolian sandy soils in Inner Mongolia. The authors documented multi-year baseline improvement and permanent enrichment of key bacterial phyla, such as *Bacteroidota* and *Gemmatimonadota*, which are foundational drivers of long-term nutrient retention. Julihaiti et al. further explored regional specificity by mapping divergent pathways that regulate soil microbial respiration. Their findings showed that baseline aridity dictates microbial metabolic responses during land enclosure and primary metabolite gatekeeper in dry soils is dissolved organic carbon. A study on the highly weathered and fragile purple soils of southwest China demonstrated that organic material mulching is a powerful biological steering mechanism under an intensive triple-cropping framework (Ren et al.). A selective upregulation of microbial consortia was observed in response to balanced carbon and nitrogen cycling, which enhanced faba bean production. Similarly, Chai et al. explored the interactive effects of maize straw incorporation and optimized irrigation in saline-alkaline soils. The integrated management technique effectively suppressed opportunistic halotolerant pathogens and reduced overall soil salinity by 53.56%.

Other studies on this Research Topic have exclusively explored “soil multifunctionality” and the temporal dynamics of soil recovery in the face of long-term application of biological amendments. Xu R. et al. investigated the mechanics of soil organic carbon (SOC) dynamics during reductive soil disinfestation. They showed that up to 75% of the newly stabilized SOC in restored systems is derived from microbial necromass, and by altering the chemical signatures of organic inputs, managers can manipulate bacterial community life-history traits. This focus was also examined by Zhao et al., who evaluated the impact of straw return on agricultural soils, showing that organic residues increase soil multifunctionality by improving microbial network complexity and connectivity. In these networks, keystone bacterial taxa serve as decentralized hubs that coordinate multistep nutrient transformations. Likewise, Li et al. examined decades-long succession and assembly of the microbiome in reclaimed farmlands in coal mining areas. A study on open-pit coal mine reconstructed ecosystems demonstrated that plant root exudates serve as critical chemical signals guiding the resumption of disrupted soil microbiota (Yang et al.). Another interesting study evaluated biocrusts in desert ecosystems, showing that cyanobacteria develop over time, expand their genetic diversity, and play a key role in transforming sand dunes into fertile topsoil (Jiang et al.).

Some studies on this Research Topic have categorically explored the potential of microbial inoculants to safely optimize traditional inputs. Such as, a liquid bioformulation based on indigenous phosphate-solubilizing *Burkholderia* and *Pseudomonas* strains delivered a 20% increase in total potato tuber yield while simultaneously reducing use of synthetic chemical phosphorus (Bansal et al.). Zhang J. et al. investigated the dual approach of combining lime and microbial agents in acidic soils. This strategy remodels the soil fungal community, suppresses soil-borne pathogens, and restores the critical soil macronutrient balance, thereby significantly reducing chemical lime inputs. The potential of zinc-solubilizing *Bacillus* strains in nutrient-deficient soils was addressed by Ahmed et al. Traditionally, the high application of zinc sulfate has caused heavy metal accumulation in agricultural soils. However, using natural zinc-mobilizing bacteria made insoluble zinc minerals bioavailable, enhancing the overall rice biomass, zinc concentrations in rice grains and chlorophyll content. At the forefront of technological innovation, Bo et al. explored the synergistic effects of combining beneficial microbial inoculants with Spent Mushroom Substrate (SMS) amendments. This mixture acted as a biological scaffold, improving soil moisture and aeration of depleted forest soil and accelerating the growth and root development of pioneer *Pinus* seedlings. In another study, Zhang H. et al. evaluated the effects of mineral bioactivation by inoculating indigenous fungi into highly toxic, nutrient-poor coal gangue-based artificial soils. To completely decode the unique biological and chemical pathways, Walia et al. applied genome-resolved metagenomics to traditional fermented biofertilizers, including *Jeevamrut* and *Beejamrut*, which validated the high-resolution blueprint of genomic pathways of carbon cycling, phytohormone synthesis, and nitrogen fixation. Advanced metabolic modeling of three novel haloalkaliphilic species of the genus *Kocuria* indicated their ubiquitous trait of phenolic acid degradation in allelochemical-infested soils (Xu L. et al.). Vu et al. proposed remediation strategies for mitigating chemical toxins in agricultural soils. They reported a novel DDT-degrading *Pseudomonas vietnamensis* T006, which can utilize highly toxic organochlorine DDT residues as a primary carbon source. An integrated multi-omics evaluation of municipal sewage sludge compost as a soil amendment was performed by Mei et al. The results indicated the enrichment of plant-beneficial taxa, including *Streptomyces* and *Mesorhizobium*, and enhanced nutrient availability in response to moderate application rates of sludge compost.

Extending the microbial solutions beyond bacteria and fungi, Mujtaba et al. evaluated two microalgal strains, *Tetradesmus nygaardii* and *Closteriopsis acicularis*, as biofertilizers for wheat and spinach. The treated plants showed enhanced biomass and chlorophyll contents and increased grain yield. Cai et al. systematically examined the synergistic regulatory mechanisms underlying the combined application of compound microbial inoculants and forage-specific fertilizers in degraded alpine mining ecosystems. A clear mitigation of soil salinity levels, enrichment of beneficial soil taxa such as *Pseudomonadota* and *Bacteroidota*, and increased soil carbon and nitrogen content were observed in response to optimized fertilizer-microbe ratios. In another study, an innovative *Stropharia rugosoannulata*–ornamental sunflower rotation system incorporating SMS was used for the synergistic remediation of deteriorated agricultural facilities. The system effectively restored soil microecological functions and provided a sustainable approach for agricultural waste valorization (Lu et al.). Finally, Lyu et al. conducted a 3-year experiment in which they evaluated composite effective microorganisms biofertilizer and organic fertilizer co-application for modulating vegetation-soil-bacteria interaction networks in artificial grasslands in alpine mining regions.

Overall, the published work on this Research Topic reaffirms that sustainable soil management and restoration strategies require holistic and comprehensive approaches, including soil microbial dynamics, agronomic practices, and systematic application of biological inputs. Furthermore, global policy frameworks must prioritize bioactive soil matrices and leverage microbial solutions to restore depleted land.

